# Autophagy Involvement in Non-Neoplastic and Neoplastic Endometrial Pathology: The State of the Art with a Focus on Carcinoma

**DOI:** 10.3390/ijms252212118

**Published:** 2024-11-12

**Authors:** Cristina Pizzimenti, Vincenzo Fiorentino, Chiara Ruggeri, Mariausilia Franchina, Alfredo Ercoli, Giovanni Tuccari, Antonio Ieni

**Affiliations:** 1 Section of Pathology, Department of Human Pathology in Adult and Developmental Age “Gaetano Barresi”, University of Messina, 98125 Messina, Italy; cristinapizzimenti86@gmail.com (C.P.); vincenzo.fiorentino@unime.it (V.F.); mariaausilia.franchina@studenti.unime.it (M.F.); 2 Section of Gynecology and Obstetrics, Department of Human Pathology in Adult and Developmental Age “Gaetano Barresi”, University of Messina, 98125 Messina, Italy; chiararug91@gmail.com (C.R.); alfredo.ercoli@unime.it (A.E.)

**Keywords:** autophagy, endometriosis, endometrial carcinoma, targeted therapies

## Abstract

Autophagy is a cellular process crucial for maintaining homeostasis by degrading damaged proteins and organelles. It is stimulated in response to stress, recycling nutrients and generating energy for cell survival. In normal endometrium, it suppresses tumorigenesis by preventing toxic accumulation and maintaining cellular homeostasis. It is involved in the cyclic remodelling of the endometrium during the menstrual cycle and contributes to decidualisation for successful pregnancy. Such a process is regulated by various signalling pathways, including PI3K/AKT/mTOR, AMPK/mTOR, and p53. Dysregulation of autophagy has been associated with benign conditions like endometriosis and endometrial hyperplasia but also with malignant neoplasms such as endometrial carcinoma. In fact, it has emerged as a crucial player in endometrial carcinoma biology, exhibiting a dual role in both tumour suppression and tumour promotion, providing nutrients during metabolic stress and allowing cancer cell survival. It also regulates cancer stem cells, metastasis and therapy resistance. Targeting autophagy is therefore a promising therapeutic strategy in endometrial carcinoma and potential for overcoming resistance to standard treatments. The aim of this review is to delve into the intricate details of autophagy’s role in endometrial pathology, exploring its mechanisms, signalling pathways and potential therapeutic implications.

## 1. Introduction

Autophagy is a cellular process crucial for maintaining homeostasis in normal tissues. Basal levels of autophagy prevent cellular accumulation of damaged proteins and organelles, which can be toxic over time [1]. By recycling intracellular components, autophagy sustains mitochondrial metabolism, promoting growth, stress tolerance and overall cellular health [2]. In response to various environmental stresses such as nutrient deficiency, hypoxia, DNA damage, and cytotoxicity, such a process is stimulated to recycle nutrients and generate energy for cell survival in unfavourable conditions [3]. Moreover, autophagy contributes to the preservation of tissue homeostasis being involved in the constitutive turnover of subcellular components in most tissues [4]. In normal tissues, autophagy mediates damage mitigation, thus suppressing tumorigenesis [5], and prevents the toxic accumulation of damaged proteins and organelles, particularly mitochondria, which can lead to oxidative stress, chronic tissue damage and oncogenic signalling [1]. It therefore inhibits the malignant transformation of cells by maintaining cellular homeostasis and normal metabolism under physiological conditions [6]. However, autophagy has been implicated in various aspects of cancer biology. In fact, its role in cancer is multifaceted and context-dependent, with different impact based on the tumour type, stage of tumorigenesis and cellular context. It has been proved that autophagy can act as both a tumour suppressor and a promoter of tumour progression [7]. Furthermore, in the early stages of cancer, it can function as a tumour suppressor by maintaining cellular homeostasis and normal metabolism, thereby inhibiting tumour initiation [8]. By contrast, in established neoplasms, autophagy can support tumour progression by promoting cancer cell survival and providing essential nutrients during the metabolic stress of cancer progression [9]. Moreover, it has been implicated in the regulation of cancer stem cells, tumour metastasis and therapy resistance [10]. Additionally, autophagy supports the survival of cancer stem cells, contributes to the maintenance of stemness and enhances resistance to anticancer therapies [11]. The complex interplay between autophagy and cancer underscores the importance of understanding the molecular mechanisms underlying autophagy in cancer progression, although it has been linked to the epithelial-to-mesenchymal transition, metastasis and the modulation of the tumour microenvironment [12]. Therefore, targeting autophagy in cancer therapy has emerged as a promising approach, with studies exploring the potential of autophagy modulation as a strategy to enhance the efficacy of anticancer treatments [13]. Overall, autophagy has garnered significant attention in the field of gynaecological pathology and specifically it has emerged as a critical player in the pathogenesis and treatment response of endometrial carcinoma. The aim of this review is to delve into the multifaceted role of autophagy in normal and hyperplastic endometrium as well as in endometrial cancer to better understand their intricate interplay.

### 1.1. Different Forms of Autophagy

There are three main types of autophagy: microautophagy, macroautophagy and chaperon-mediated autophagy (CMA) [14,15]. Microautophagy and macroautophagy are two distinct forms of autophagy that involve different mechanisms for the degradation of cellular components. In microautophagy, cellular cargo is directly engulfed by the lysosome through invagination of the lysosomal membrane. This process involves the direct uptake of cytoplasmic material by the lysosome for degradation [16]. Conversely, macroautophagy involves the formation of double-membraned vesicles called autophagosomes that sequester cytoplasmic components, such as organelles or proteins. These autophagosomes then fuse with lysosomes to form autolysosomes, where the cargo is degraded by lysosomal enzymes [17]. Both microautophagy and macroautophagy can be upregulated in response to nutrient deprivation, oxidative stress or cellular damage. However, macroautophagy involves a series of complex signalling pathways and protein interactions to initiate and complete autophagosome formation [17]. Basically, microautophagy is involved in the maintenance of cellular quality control and plays a role in basal autophagic activity in cells [16]. Conversely, macroautophagy is a major pathway for bulk degradation of cellular components, including damaged organelles, protein aggregates and pathogens, and uses autophagosomes for selective degradation of cargo; this latter requires both assembly of autophagosome and autophagy receptors, which are needed for recognising target proteins. On other hand, CMA is a more selective form of autophagy in which specific proteins are targeted for degradation [18]. These proteins contain a pentapeptide motif (KFERQ) in their amino-acid sequence that is recognised by a specific chaperone protein named cytosolic chaperone HSPA8 (heat shock protein family A [HSP70] member 8) that delivers them into the lysosomal lumen for degradation; the interaction with LAMP2 (lysosomal-associated membrane protein 2) is required to facilitate lysosomal membrane translocation [18].

### 1.2. Molecular Mechanism and Regulation Pathways of Autophagy

The molecular mechanisms of autophagy are complex and involve various signalling pathways, including the PI3K/AKT/mTOR pathway, the AMPK-mTOR pathway and the p53 pathway [19]. These pathways regulate the expression and activity of autophagy-related genes (ATGs), which are essential for the different steps of the autophagic process. In fact, the autophagy mechanism consists of five phases, which are controlled by several autophagy-related proteins (ARPs) [14]. The first phase is called “initiation”, and it is controlled by the AMP-activated protein (AMPK)/mammalian target of rapamycin (mTOR) signalling pathway [19]. Under stressful conditions, an increase in AMPK inhibits mTOR, thus leading to the formation of the autophagy initiation complex formed by ULK1/2, Atg13, Atg10 and FIP200 [19,20,21]. These proteins promote the recruitment of the autophagy nucleation complex [20,21]. The second phase of autophagy is called “nucleation”, and it is regulated by another protein complex formed by the Beclin-1/class-III PI3K (PI3K-III) complex in association with other ATGs, such as UVRAG and AMBRA-1 [22,23]. The “elongation” phase is regulated by two ubiquitin-like protein systems [24,25]. The first complex, formed by the ARPs Atg12, Atg5 and Atg16, mediates the binding of LC3 protein on the outer side of the autophagosome, whereas the other complex, formed by phosphatidylethanolamine (PE), microtubule-associated protein 1 light chain 3 (MAP1LC3), is cleaved by Atg4B into LC3-I exposing a reactive glycine residue for the binding with Atg7 and ATG and the formation of LC3-II. Thus, in the “completion” and “fusion” phases, LC3-II promotes the closure of the autophagosome and the fusion with the lysosome for the degradation of substrates [26]. These substrates are recruited by p62 or sequestosome-1 (p62/SQSTM1) protein that is a carrier protein that binds ubiquinated substrates and forms aggregates that are recognised by LC3-II on the inner side of the autophagosome [27]. Finally, lysosomal enzymes degrade the damaged proteins, and their components can be recycled and returned to the cytosol [14]. As already mentioned, the AMPK/mTOR signalling pathway is the main regulator of the autophagic process [28,29]. In physiological conditions, mTOR is active and inhibits autophagy initiation through the phosphorylation of the ULK1/2 complex.

Further signalling pathways, such as PI3K/AKT/mTOR (often activated through mutations in PIK3CA or loss of PTEN) and MAPK/ERK, are also involved in autophagy regulation [30]. For example, the activation of MAPK/ERK promotes the inhibition of mTOR and the increase in Beclin-1 activity, whereas PI3K is involved in the inhibition of autophagy through the activation of AKT [31]. In addition, several different transcription factors are involved in autophagy regulation. The dephosphorylation of the transcription factor EB (TFEB) promotes the activation of several ATGs, such as BECN1, ATG9B, MAP1LC3B, ATG5, SQSTM1 and UVRAG [32]. Moreover, the translocation to the nucleus of the Forkhead box class O (FoxO) family of transcription factors induces the expression of several ATGs involved in the different phases of the autophagy mechanism (e.g., ULK, Beclin-1, ATG14, MAP1LC3B, ATG4, TFEB) [33]. Under stressful conditions, such as hypoxia, transcription factor hypoxia-inducible Factor-1 (HIF-1) is activated and induces the expression of BNIP3, BNIP3L, Beclin-1 and Atg5, regulating autophagy, cell proliferation and survival and angiogenesis [34].

## 2. Autophagy in Normal Endometrium and Benign Endometrium-Related Conditions

**Figure 1:**
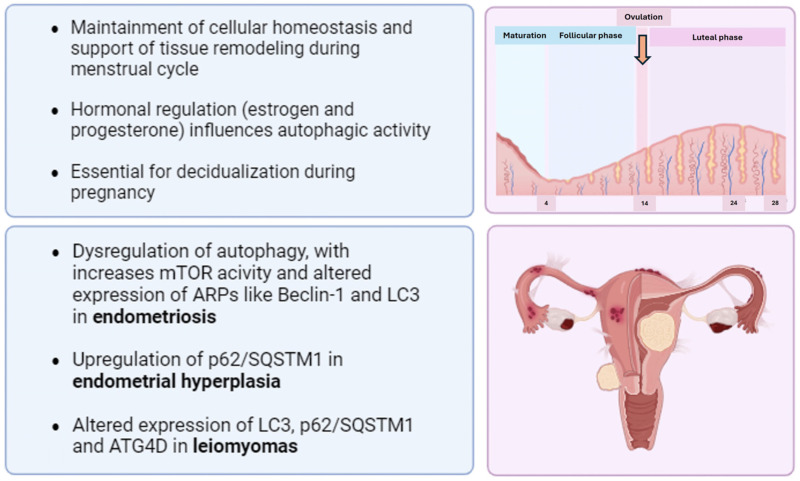
Mechanisms of autophagy flux in normal, hyperplastic and dysfunctional uterine pathology (the figure was created with BioRender.com, accessed on 21 August 2024).

In normal endometrium, autophagy plays a significant role in physiological processes, contributing to cellular homeostasis, tissue remodelling and response to hormonal changes. In fact, it is involved in the cyclic changing of the endometrium during the menstrual cycle [35], and an increased autophagosome formation has been observed during the secretory phase, indicating a role of autophagy in preparation for menstruation and tissue removal (Figure 1). Interestingly, the expression of LC3B-II, already considered as an indicator of autophagic activity, is elevated in the secretory phase of normal endometrial tissues [36]. Moreover, the activation of autophagy in endometrial glandular cells during menstruation and tissue regeneration is documented by the presence of autophagy markers such as LC3A [35]. Overall, an increased autophagic activity has been observed as the cycle progresses [37,38,39], and it has been shown that the induction of such a process is closely linked to apoptosis, highlighting its role in regulating cell death processes in the endometrium [37]. Obviously, hormonal control is essential in modulating autophagic activity during the menstrual cycle: in fact, autophagy is low in the proliferative phase, dominated by oestrogen, but increases in the secretory phase, dominated by progesterone. The withdrawal of these hormones during menstruation triggers a peak in autophagy, facilitating the shedding of the endometrial lining and subsequent tissue repair [38,40]. Moreover, autophagy contributes to the decidualisation process in the endometrium, which is essential for a successful pregnancy [40]. For instance, the downregulation of autophagy has been associated with impaired decidualisation of endometrial stromal cells, impacting endometrial physiology [41].

Taking into consideration the role of autophagy in some benign endometrial-related conditions, an aberrant autophagic flux has been observed in conditions like endometriosis (Figure 1), where dysregulation of ARPs has been detected [42], and it has also been linked to the pathogenesis of such condition [43]. In particular, some early studies showed that ectopic endometrial cells presented low levels of Beclin-1 compared to endometrial cells of patients without endometriosis [44]. Moreover, Beclin-1 was negatively correlated with serum CA-125 level and pelvic pain, suggesting that low expression of Beclin-1 could contribute to the development of endometriosis [44,45]. In addition, a recent study suggests that decreased levels of Beclin-1 and LC3 may be related to the occurrence and development of endometriosis [46]. On the contrary, another study reported that autophagy protein levels such as LC3 and Beclin-1 are upregulated in ovarian endometriosis [47]. Interestingly, it has been shown that mTOR may play a role in the pathogenesis of endometriosis [40] (Figure 1). In fact, as already discussed, it acts as a negative regulator of autophagy, and its activity has been found to be abnormally increased in endometriosis. This increased mTOR activity can lead to the suppression of autophagy, which may contribute to the survival and growth of endometrial cells outside the uterus. As a proof of this, the inhibition of mTOR by pharmacological agents, such as rapamycin, has been shown to induce autophagy and promote the death of endometriotic cells [40].

The role of autophagy in endometrial hyperplasia (EH) is still mostly unknown, with just two studies that showed a correlation between upregulated expression of p62/SQSTM1 and the promotion of EH [48] (Figure 1). However, it has been reported that treatment with metformin (a biguanide antidiabetic drug) or sorafenib (a kinase inhibitor drug) upregulates the expression of LC3 and downregulates the protein level of p62/SQSTM1, thus decreasing the risk of EH in polycystic ovarian syndrome (PCOS) through the activation of the mTOR complex [49]. Finally, expressions of ARPs including LC3, p62/SQSTM, and ATG4D may result in a dysregulation of autophagy, an important contributor to the development of stromal uterine leyomiomas [50,51] (Figure 1); in detail, a decreased expression of ATG4D has been shown to promote the growth of leyomiomas [50].

## 3. The Role of Autophagy in Endometrial Carcinoma

Endometrial carcinoma is a malignant tumour originating from the epithelial lining of the uterus and representing the sixth most frequent cancer in women between 50 and 69 years old with a worldwide incidence that has increased by more than 130% within the past 30 years [52]. Furthermore, endometrial carcinoma has been commonly classified based on histological features into type I and type II cancer [52]. The type I group comprises 80% of all endometrial cancers of endometrioid origin: they are often associated with oestrogen exposure and endometrial hyperplasia, have generally a most favourable prognosis and are divided in subtypes such as mucinous, villoglandular and secretory adenocarcinomas [52]. The type II group is composed of non-endometrioid carcinomas, such as serous carcinomas, clear cell carcinomas and carcinosarcomas: they are less common but present a more aggressive behaviour compared to type I carcinomas [52,53,54,55]. They are oestrogen-independent neoplasms, often chemoresistant, usually detected at a higher stage and therefore associated with a poor prognosis [53,54,55]. In 2013, The Cancer Genome Atlas Research Network (TCGA) published a new classification based on the molecular features of endometrial carcinoma. This classification subdivided endometrial carcinomas in four groups: (1) POLE ultra-mutated, characterised by an extremely high number of mutations due to defects in the POLE gene and generally associated with a good prognosis; (2) microsatellite instability (MSI) hypermutated, characterised by high frequency of mutations in short DNA sequences (microsatellites), often linked with Lynch syndrome, with an intermediate prognosis; (3) copy-number low (CNL), involved in most low-grade endometrioid carcinomas and characterised by low frequency of DNA copy number alterations and favourable prognosis; (4) copy-number high (CNH), that includes serous and high-grade endometrioid carcinomas, characterised by high frequency of DNA copy number alteration and poor prognosis [56].

Nowadays, the management of endometrial carcinoma is based on the specific features of the tumour, such as stage, histological and molecular subtype and patient’s characteristics including age, comorbidities and desire for future fertility [57]. The surgical approach is indicated in cases of cancer localised to the uterus and involves total hysterectomy with bilateral salpingo-oophorectomy, lymphadenectomy and peritoneal washing. In high-risk tumours, adjuvant therapy is recommended after the surgical approach. In particular, radiotherapy is utilised for early-stage or high-risk tumours, whereas chemotherapy is recommended for advanced tumours, including those with a high-grade histology that spread beyond the uterus. Carboplatin, or paclitaxel, is usually used alone or in combination with radiotherapy. Hormone therapy is recommended for early-stage and/or low-grade tumours, and target therapy, including immune system checkpoint inhibitors, is used for recurrent or advanced endometrial carcinomas with specific molecular features, such as mismatch repair deficiency [57].

**Figure 2:**
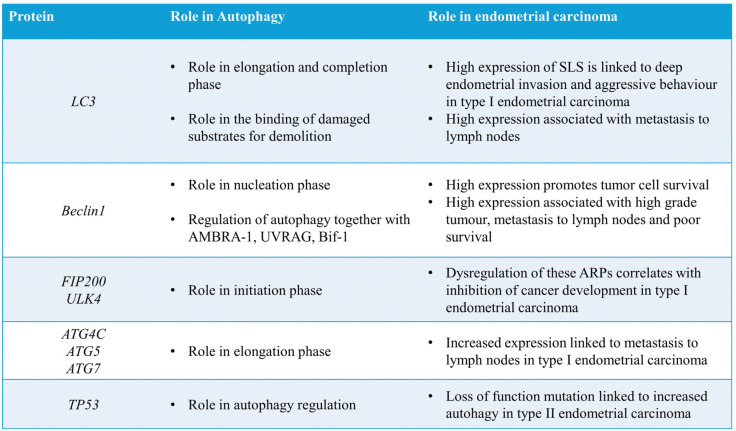
The main autophagic-related proteins involved in endometrial carcinoma.

The pathogenetic role of autophagy in endometrial carcinoma is a complex and multifaceted process that involves intricate molecular mechanisms influencing tumour initiation, progression and response to therapy (Figure 2). Autophagy plays a dual role in endometrial cancer, influencing both tumour progression and treatment response (Figure 2). Studies have highlighted the complex relationship between autophagy and chemosensitivity in endometrial carcinoma cells during chemotherapy [58].

In type I endometrial carcinomas, the PI3K/Akt/mTOR pathway exhibits a hyperactivation, leading to inhibition of autophagy induction that appears to be related to cell proliferation and survival [59]. In detail, a specific pattern of LC3 reactivity called “stone-like structures” (SLS) is linked to excessive autophagy activity, to deep endometrial invasion and to a more aggressive behaviour of the tumour [59]. In addition, SLS expression appears to be higher in endometrial carcinoma compared to atypical endometrial hyperplasia and normal endometrial tissue [59]. Moreover, it has been reported that higher levels of Beclin-1 are associated with high-grade tumours, deep myometrial invasion and a poor five-year survival [59]. However, a hypoxic microenvironment and high expression of Beclin-1 increase autophagy and promote tumour cells’ survival [59].

It has been revealed that autophagy induction is also controlled by Oestrogen Induced Gene-121 (EIG121), a transmembrane protein reported and overexpressed in endometrial dysplasia and suppressed in oestrogen-dependent endometrial carcinoma [60]. Moreover, Lebovitz et al. [61] reported an association between alterations in autophagic genes, such as ATG4C, RB1CC1/FIP200 and ULK4, and endometrial type I carcinomas that results in a reduction of autophagy and an inhibition of cancer development [61]. Interestingly, an increased expression of Beclin-1, ATG5, ATG7 and LC3 has been related to the development of metastatic endometrial carcinoma to the lymph nodes [62]. Moreover, it has been shown that mutations in the tumour suppressor gene TP53 play significant role in autophagic processes [63]. TP53 is a critical tumour suppressor gene essential for genomic stability and regulation of cell cycle progression, and loss-of-function mutations in such a gene can result in increased autophagy through p16 induction, which has been associated with type 2 endometrial carcinogenesis [63]. Likewise, it has also been shown that depending on its cellular localisation and the type of stress, p53 can either induce or inhibit autophagy [64].

An altered autophagic flux in endometrial carcinoma can be also triggered by several treatment strategies. For example, calorie restriction promotes an increase in autophagy rates that reduces the growth of endometrial lesion such as endometriosis in mice, although its efficacy in endometrial carcinoma has not been yet determined [65]. Furthermore, combined oral contraceptives have been reported to suppress autophagy activity in mice, reducing the risk of endometrial carcinoma [66], and to attenuate BECN1 mRNA expression in the ectopic endometrium of patients with endometriosis [67]. Also, metformin has shown activity in promoting autophagy through the activation of AMPK and the inactivation of mTOR [49,68]. High expression of ATGs such as ATG3, LC3 and p62/SQSTM1 has been found in normal endometrium of PCOS patients after metformin treatment compared to the low expression in non-treated PCOS patients [49,68,69]. Resistance mechanisms include reactivation of mTOR signalling through compensatory mechanisms or activation of downstream effectors [70]. Another autophagy inhibitor is chloroquine, a lysosomotropic agent that inhibits autophagy by preventing lysosomal acidification and autophagosome–lysosome fusion [70,71]. It has been shown that it reduces the growth of endometrial tumour cells resistant to cisplatin [71]. Resistance can arise through the upregulation of lysosomal biogenesis or the activation of alternative degradation pathways like the ubiquitin–proteasome system [72]. Moreover, cisplatin has been reported to enhance autophagy activity through the activation of the PI3K/AKT/mTOR pathway, thus promoting endometrial carcinoma resistance to treatment [73]. An increase in autophagy activity has been also observed in carcinoma resistant to treatment with paclitaxel [74].

## 4. Autophagy and Carcinoma Stem Cells

Cancer stem cells (CSCs) are a small subpopulation of cells within tumours that possess self-renewal and differentiation capabilities, contributing to tumour initiation, progression and recurrence [75]. It has been shown that autophagy plays a dual role in CSCs, either promoting or inhibiting their survival and function depending on the cellular context and stress conditions [75]. In detail, under stress, autophagy can promote CSC survival by providing energy and nutrients through the degradation of cellular components [75]. However, excessive or prolonged autophagy can also lead to cell death [75]. In endometrial carcinoma, recent research has revealed a significant role for autophagy in the regulation of endometrial cancer stem cells (ECSCs) [76]. In fact, higher autophagy levels have been observed in ECSCs compared to non-CSCs, suggesting that autophagy may be crucial for maintaining stemness in EC cells, a phenomenon which has been positively correlated with autophagy levels [76]. Moreover, EIG121, a protein involved in autophagy regulation, has been identified as a potential dual regulator of autophagy and stemness in endometrial carcinoma cells [76]. Increased expression of EIG121 was found to enhance both autophagy and stemness in ECSCs, highlighting the potential link between these two processes. This finding suggests that EIG121 may be a promising therapeutic target for endometrial carcinoma, as its inhibition could potentially reduce both autophagy and stemness in ECSCs [76]. Furthermore, the expression of Musashi-1, an adult stem cell marker, has been linked to endometriosis and endometrial carcinoma, since Musashi-1-expressing cells have been found in proliferating endometrium and neoplastic endometrial cells [77]. In addition, the modulation of cell cycle progression and apoptosis in endometrial carcinoma cells regulated by Musashi-1 highlights its potential role in stemness-related factors, like Notch-1 and p21 WAF1/CIP1, presenting a further reliable target for novel therapeutic interventions [78]. Overall, the complex relationship between autophagy and CSCs in endometrial carcinoma presents both challenges and opportunities for therapeutic intervention. Therefore, a more nuanced approach that targets specific autophagy-related proteins or pathways may be necessary to effectively eliminate ECSCs without promoting their survival.

## 5. Resistance to Treatment and Autophagy Targeting Agents

Autophagy has been implicated in promoting chemoresistance in endometrial cancer cells [79]. In fact, activation of autophagy in response to chemotherapy agents can enhance cell survival and resistance to treatment [79]. However, the upregulation of autophagy as a protective mechanism against chemotherapy-induced stress poses a significant challenge in the effective management of endometrial cancer (Table 1). For example, cisplatin and paclitaxel, two commonly used chemotherapeutic agents for endometrial carcinoma, have been shown to induce autophagy in endometrial carcinoma cells [79]. This autophagy induction can protect the cancer cells from therapy-induced cell death, leading to treatment resistance. On the other hand, progestin therapy has been considered another treatment option for endometrial carcinoma, but resistance to progestins is a significant clinical challenge in which autophagy has been implicated [79]. Given the role of autophagy in chemoresistance, its inhibition has emerged as a potential therapeutic strategy to enhance the efficacy of chemotherapy in endometrial carcinoma [79]. Studies have shown that combining autophagy inhibitors, such as chloroquine, with cisplatin or paclitaxel can increase the sensitivity of endometrial carcinoma cells to these drugs and improve treatment outcomes [58]. Furthermore, the autophagy inhibition of 3-Methyladenine (3-MA) results in the downregulation of L-type calcium channel a1D subunit Cav1.3 and enhances cell death induced by nitrendipine [80].

While autophagy inhibition shows promise in overcoming treatment resistance in endometrial carcinoma, it is noteworthy that autophagy can also have pro-death effects in cancer cells and, therefore, also autophagy inducers have been studied. In fact, mTOR inhibitors like everolimus and temsirolimus can induce autophagy, leading to tumour cell death in some cases, and showing promise in preclinical and clinical studies of endometrial carcinoma [80]. In particular, temsirolimus showed higher sensitivity in high-grade tumours compared to cisplatin, doxorubicin and paclitaxel [81], and in a phase II clinical study, the combined use of everolimus and letrozole resulted in a high ORR in patients with recurrent EC [82]. Another agent targeting the PI3K/AKT/mTOR pathway is ABTL08212, a fatty acid-derived molecule that has been evaluated as an alternative first-line treatment for IK, AN3CA and HEC-1A endometrial cancer cell lines. ABTL08212 promotes the activation of autophagy and apoptosis through the downregulation of mTOR and the transformation of LC3-I into LC3-II [83]. However, the efficacy of mTOR inhibitors as single agents is limited, and combination therapies with other agents may be necessary to achieve optimal results. Additionally, inhibiting the PI3K/AKT/mTOR pathway has been shown to re-sensitise progestin-resistant endometrial carcinoma cells to progestins [84]. Furthermore, second-generation mTOR inhibitors have been developed, targeting catalytic sites of mTOR complexes; for example, mTORC1/2 inhibitors AZD8055 and OSI-027 have been demonstrated to inhibit EC cell growth in mice models [30,85].

Autophagy activators such as rapamycin can increase apoptosis in EC cells induced by cisplatin, exhibiting a synergistic effect with cisplatin [86]. Moreover, metformin has been shown to overcome progesterone resistance in endometrial carcinoma and to promote cell apoptosis through the increase in expression of LC3 and Beclin-1 [70]. Recently, it has been demonstrated that glucagon-like peptide-1 receptor (GLP-1R) agonist liraglutide activates autophagy through the AMPK pathway in EC cells, characterised by high expression of LC3 and low levels of p62 [87]. Moreover, MHY2256, an inhibitor of sirtuin protein (SIRT), promotes apoptosis and reduces tumour growth in EC cells of mice models through the increase in LC3-II and ATG5 levels [88]. Finally, it has been shown that some therapeutic agents activate autophagy through the promotion of endoplasmic reticulum (ER) stress [89]. In particular, SI113, an inhibitor of SGK1 (a serine/threonine protein kinase that controls ER stress), promotes the activation of autophagy and the increase in LC3 and Beclin-1 levels in EC cells [89]. Overall, the development of novel autophagy-targeting agents with improved efficacy and safety profiles is an active area of research, suggesting that therapeutic targeting of autophagy in endometrial carcinoma may represent a promising and challenging field. These agents may target specific molecules or pathways involved in autophagy regulation, such as ULK1/2, ATG5, or Beclin-1 [79]. In future studies, it would be desirable to identify reliable biomarkers that predict response to autophagy modulation for personalised therapeutic approaches in endometrial carcinoma.

**Table 1:** Role and action of different proposed drugs related to autophagy in endometrial carcinoma.

Drug	Direct/Indirect Action on Autophagy	Mechanism of Action
Metformin [49,70]	Indirect	Activates autophagy through AMPK and inactivation of mTOR
Paclitaxel [58,74,81]	Indirect	Increases autophagy activity
Combined oral contraceptives [66]	Indirect	Suppress autophagy activity
Chloroquine [71,72]	Direct	Inhibits autophagy by preventing lysosomal acidification and autophagosome–lysosome fusion
Cisplatin [73,86]	Indirect	Enhances autophagy activity through the activation of PI3K/AKT/mTOR pathway
3-Methyladenine (3-MA) [80]	Direct	Inhibits autophagy, leading to the downregulation of L-type calcium channel α1D subunit Cav1.3 and enhancing cell death induced by nitrendipine
Temsirolimus [80,81]	Indirect	mTOR inhibitor, induces autophagy leading to tumour cell death
Everolimus [82]	Indirect	mTOR inhibitor, induces autophagy leading to tumour cell death
ABTL0812 [83]	Indirect	Activates autophagy and apoptosis through the downregulation of mTOR
AZD8055 [85]	Indirect	mTORC1/2 inhibitor, inhibits EC cell growth
OSI-027 [85]	Indirect	mTORC1/2 inhibitor, inhibits EC cell growth
Rapamycin [86]	Direct	Autophagy activator, increases apoptosis in EC cells induced by cisplatin
Liraglutide [87]	Indirect	GLP-1R agonist, activates autophagy through AMPK pathway
MHY2256 [88]	Indirect	SIRT inhibitor, promotes apoptosis and reduces tumour growth by increasing LC3-II and ATG5 levels
SI113 [89]	Indirect	SGK1 inhibitor, promotes autophagy activation and increases LC3 and Beclin-1 levels

## 6. Conclusions

Autophagy plays a multifaceted role in endometrial carcinoma, influencing tumour progression, treatment response and therapeutic outcomes. Targeting such a biological process may offer a promising approach to enhance the efficacy of chemotherapy and overcome treatment resistance. However, a deeper understanding of its context-dependent roles and the development of personalised approaches are essential for maximising its clinical benefit. Therefore, further research into its molecular mechanisms is essential for advancing precision medicine approaches and developing targeted therapies that exploit the dual nature of such a process in endometrial carcinoma.

## Abbreviations

AMPK (AMP-activated protein), ARPs (autophagy-related proteins), ATGs (autophagy-related genes), CMA (chaperon-mediated autophagy), CNL (copy-number low), CNH (copy-number high), CSCs (cancer stem cells), ECSCs (endometrial cancer stem cells), EH (endometrial hyperplasia), EIG121 (Oestrogen Induced Gene-121), ER (endoplasmic reticulum), FoxO (Forkhead box class O), GLP-1R (glucagon-like peptide-1 receptor), HIF-1 (transcription factor hypoxia-inducible Factor-1), HSPA8 (heat shock protein family A [HSP70] member 8), KFERQ (pentapeptide motif), LAMP2 (lysosomal-associated membrane protein 2), 3-MA (3-Methyladenine), mTOR (mammalian target of rapamycin), PI3K-III (class-III PI3K), PE (phosphatidylethanolamine), PCOS (polycystic ovarian syndrome), MAP1LC3 (microtubule-associated protein 1 light chain 3), MSI (microsatellite instability), p62/SQSTM1 (p62 or sequestosome-1 protein), SIRT (inhibitor of sirtuin protein), SLS (stone-like structures), TFEB (transcription factor EB), TCGA (Cancer Genome Atlas Research Network).
